# Circulating tumor DNA shows variable clonal response of breast cancer during neoadjuvant chemotherapy

**DOI:** 10.18632/oncotarget.21198

**Published:** 2017-09-23

**Authors:** Ji-Yeon Kim, Donghyun Park, Dae-Soon Son, Seok Jin Nam, Seok Won Kim, Hae Hyun Jung, Yeon Jeong Kim, Gahee Park, Woong-Yang Park, Jeong Eon Lee, Yeon Hee Park

**Affiliations:** ^1^ Division of Hematology-Oncology, Department of Internal Medicine, Samsung Medical Center, Sungkyunkwan University School of Medicine, Seoul 06351, Korea; ^2^ Samsung Genome Institute, Samsung Medical Center, Seoul 06351, Korea; ^3^ Department of Surgery, Samsung Medical Center, Sungkyunkwan University School of Medicine, Seoul 06351, Korea; ^4^ Department of Health Sciences and Technology, Samsung Advanced Institute for Health Sciences and Technology, Sungkyunkwan University, Seoul 06351, Korea; ^5^ Department of Biomedical Sciences, Seoul National University College of Medicine, Seoul 03080, Korea; ^6^ Biomedical Research Institute, Samsung Medical Center, Sungkyunkwan University, Seoul 06351, Korea

**Keywords:** circulating tumor DNA, neoadjuvant chemotherapy, breast cancer

## Abstract

Circulating tumor DNA (ctDNA) correlates with tumor burden and provides early detection of treatment response and tumor genetic alterations in breast cancer (BC). In this study, we aimed to identify genetic alterations during the process of tumor clonal evolution and examine if ctDNA level well indicated clinical response to neoadjuvant chemotherapy (NAC) and BC recurrence.

We performed targeted ultra-deep sequencing of plasma DNAs, matched germline DNAs and tumor DNAs from locally advanced BC patients. Serial plasma DNAs were collected at diagnosis, after the 1^st^ cycle of NAC and after curative surgery. For the target enrichment, we designed RNA baits covering a total of ∼202kb regions of the human genome including a total of 82 cancer-related genes.

For ctDNA, 15 serial samples were collected and 87% of plasma SNVs were detected in 13 BC samples that had somatic alterations in tumor tissues. The *TP53* mutation was most commonly detected in primary tumor tissues and plasma followed by *BRCA1* and *BRCA2*. At BC diagnosis, the amount of plasma SNVs did not correlate with clinical stage at diagnosis. With respect to the therapeutic effects of NAC, we found two samples in which ctDNA disappeared after the 1^st^ NAC cycle achieved a pathologic complete response (pCR). In addition, the amount of ctDNA correlated with residual cancer volume detected by breast MRI.

This targeted ultra-deep sequencing for ctDNA analysis would be useful for monitoring tumor burden and drug resistance. Most of all, we suggest that ctDNA could be the earliest predictor of NAC response.

## INTRODUCTION

Locally advanced breast cancer (LABC) is defined as breast cancer (BC) larger than 5 centimeters or with lymph node metastasis [1]. Usually, LABC is treated with neoadjuvant chemotherapy (NAC) followed by curative surgery to reduce tumor size and eliminate micrometastasis [2]. Response to NAC helps predict BC prognosis [3]. Pathologic complete response (pCR), defined as no residual tumor cells after NAC, represents prolonged survival without BC recurrence and residual cancer burden score, based on residual tumor volume, and can more accurately predict BC outcomes [4]. However, this clinical information does not provide biological information about BC that can be applied to BC treatment.

Primary tumor biopsy is performed to determine biological characteristics related to prognosis; serial biopsy of BC specimens can identify pathological changes under NAC. Moreover, DNA alteration and mRNA expression analysis using serial tumor specimens indicating changes in tumor biology and alterations in somatic mutations have been experimentally studied [5]. Ki-67, a protein marker of BC proliferation, decreases in BC with good prognosis after one cycle of NAC compared to BC with poor prognosis. In addition, the apoptotic index after paclitaxel chemotherapy is an early predictor of disease response [6, 7]. Tumor-associated lymphocytes are regarded as another independent predictor of NAC [8]. Because NAC has a role in good research conditions as above, NAC condition could provide a great environment for window-of-opportunity trials [9].

Recently, liquid biopsy has replaced tumor biopsy because of its high feasibility and easy access. Circulating tumor DNA (ctDNA), which is circulating free DNA in the blood that originates from cancers, can be detected by recently-developed technologies. CtDNA could facilitate early disease detection, diagnosis and detection of disease recurrence. CtDNA also provides a genomic profile of BC and predicts drug response [10, 11]. In BC, ctDNA correlates with tumor burden and provides early detection of treatment response and tumor genetic alterations [12]. In addition, the amount of ctDNA in early stage BC can be used to monitor NAC response and serial follow-up of ctDNA supports earlier detection of tumor recurrence by about 2 months compared to clinical methods for detection tumor recurrence [13].

In this study, we aimed to identify the correlations in genomic profile and clonal evolution between tumors and ctDNA during NAC. We investigated changes in tumor and ctDNA genetic information that affected the clinical response to NAC and BC recurrence.

## RESULTS

### Patient characteristics

A total of 20 patients were enrolled (Supplementary Figure 1). CtDNA were collected from only 15 patients because one patient withdrew before first sampling, one patient was diagnosed as breast angiosarcoma and three ctDNA samples were of poor quality. Because of patient withdrawal and missing specimens, only 9 pairs of tumor tissues were collected.

Clinical characteristics are presented in Table 1 : 11 tumors were triple-negative breast cancers (TNBCs), 2 were estrogen receptor (ER)-positive and 2 were HER2-positive BCs; 5 were diagnosed as stage IIB at diagnosis, 6 were IIIA, 1 was IIIB and 3 were IIIC. All BCs were treated using adrimycin/cyclophosphamide combination chemotherapy and taxane-based chemotherapy. Trastuzumab was used for HER2-positive BC treatment. After NAC, 3 cases of TNBC showed pathological complete response (pCR) and the other 12 had residual tumors.

**Table 1:** Patient characteristics

No.	Subtype	Clinical Stage	Neoadjuvant chemotherapy	Surgical stage	RCB^h^ score	RCB class
4	TNBC ^a^	3A	Paclitaxel*12->AC^d^*4	1	1.333	1
8	TNBC	3A	AC*4 -> D^e^*4	0 (pCR^g^)	0	0
9	ER^b^-HER2^c^+	3C	AC*4 -> DH^f^*4	2A	1.315	1
11	TNBC	2B	AC*4 -> D*4	1	1.630	2
13	TNBC	2B	Paclitaxel*12->AC*4	1	0.748	1
14	ER+HER2-	3C	AC*4 -> D*4	3A	2.559	2
20	TNBC	3C	AC*4 -> D*4	1	2.132	2
21	ER+HER2-	2B	AC*4 -> D*4	1	1.797	2
23	TNBC	3B	AC*4 -> D*4	3A	4.090	3
24	ER-HER2+	3A	AC*4 -> DH*4	2B	3.922	3
28	TNBC	2B	AC*4 -> D*4	2A	1.675	2
29	TNBC	3A	Paclitaxel*12->AC*4	2B	3.428	3
32	TNBC	2B	AC*4 -> D*4	2B	3.310	3
34	TNBC	3A	AC*4 -> D*4	0	0	0
35	TNBC	3A	AC*4 -> D*4	0	0	0

a: triple-negative breast cancer; b: estrogen receptor; c: human epidermal receptor 2; d: adriamycin+cyclophosphamide; e: docetaxel; f: docetaxel+herceptin; g: pathologic complete response; h: residual cancer burden.

### Circulating tumor DNA

We analyzed mutation profiles of plasma DNA compared to primary tumor tissues using an 82 cancer-related gene panel (Supplementary Table 1). Primary tumors, white blood cells and plasma DNA were sequenced using the same pipeline (Supplementary Table 2). After deduplication, the median depths of unique coverage were 1186.9× for primary tumor, 2404.5× for WBC and 2289.0× for plasma DNA.

The number of mutations in baseline primary tumors was 33 (Table 2 and 3). Of 15 BCs, 13 had more than one somatic alteration in the primary tumor tissue. We assessed whether the mutations found in primary tumor tissue samples were detectable in pretreatment plasma samples obtained before NAC treatment. The individual mutations were statistically tested to evaluate the significance of the presence of the mutations in baseline plasma DNAs [14]. Among the 33 mutations found in the 13 tumor samples, 23 mutations were significantly present above the background in the plasma DNAs, resulting in a 69.7% detection sensitivity at the variant level. We also profiled genetic variants in pretreatment plasma DNA without the information on mutations in primary tumors, which detected 17 variants with a 51.5% sensitivity. Plasma variants were 23 (69.7%) with baseline primary tumor information and 17 (51.5%) without baseline primary tumor information (Table 2). Similar to primary tissue, plasma SNVs were detected in 13 BCs that had somatic alterations in tumor tissues (86.7%). The median number of plasma SNVs was 3.92 (range 1–9).

**Table 2:** Baseline detection sensitivity

		Mutation in baseline primary tissue	Plasma variants (with primary info)	Plasma variants (without primary info)
Baseline	Pre-treatment SNVs	33	23	17
	Sensitivity (%)		69.7%	51.5%

**Table 3:** Thirty-three genes mutated in baseline tumor samples

Samples	Chr	Start	End	Ref	Alt	Gene	Primary_Depth	Primary_ReadCount	Primary_Allele Frequency
BR11	chr13	32944538	32944538	G	T	BRCA2	991	154	0.15
BR11	chr17	7578290	7578290	C	G	TP53	599	61	0.10
BR13	chr1	27023535	27023535	C	G	ARID1A	808	21	0.02
BR20	chr17	7577511	7577511	A	G	TP53	275	211	0.76
BR21	chr12	25380213	25380213	A	T	KRAS	864	19	0.02
BR21	chr17	29556190	29556190	C	T	NF1	706	44	0.06
BR21	chr17	7578235	7578235	T	C	TP53	479	227	0.47
BR23	chr17	7578208	7578208	T	C	TP53	733	562	0.76
BR23	chr3	178952085	178952085	A	T	PIK3CA	2046	1033	0.50
BR23	chr5	149435640	149435640	G	C	CSF1R	868	23	0.02
BR24	chr13	32906729	32906729	A	C	BRCA2	849	296	0.34
BR24	chr17	37881000	37881000	G	T	ERBB2	957	542	0.56
BR24	chr6	117622184	117622184	G	C	ROS1	936	393	0.41
BR24	chr6	117622188	117622188	T	G	ROS1	932	385	0.41
BR24	chr6	117622233	117622233	C	T	ROS1	1009	453	0.44
BR24	chr7	100417377	100417377	C	T	EPHB4	175	93	0.53
BR24	chr7	116340263	116340263	C	G	MET	1386	677	0.48
BR24	chr7	148525904	148525904	C	G	EZH2	657	268	0.40
BR28	chr11	108159732	108159732	C	T	ATM	1377	633	0.45
BR28	chr12	25398284	25398284	C	A	KRAS	1385	66	0.04
BR28	chr13	32906480	32906480	A	C	BRCA2	936	441	0.47
BR28	chr13	32911463	32911463	A	G	BRCA2	1246	578	0.46
BR28	chr7	55229255	55229255	G	A	EGFR	1381	613	0.44
BR28	chr9	98211572	98211572	T	A	PTCH1	704	337	0.47
BR29	chr10	89717774	89717774	A	T	PTEN	961	122	0.12
BR29	chr17	7577570	7577570	C	A	TP53	579	51	0.08
BR29	chr3	178952085	178952085	A	G	PIK3CA	1474	213	0.14
BR32	chr17	7577538	7577538	C	T	TP53	564	186	0.32
BR34	chr17	7574003	7574003	G	A	TP53	333	35	0.10
BR4	chr17	41246709	41246709	G	C	BRCA1	1013	649	0.64
BR4	chr17	7578263	7578263	G	A	TP53	713	506	0.70
BR8	chr17	7577568	7577568	C	A	TP53	482	200	0.41
BR8	chr9	93650837	93650837	G	A	SYK	2462	52	0.02

**Figure 1:**
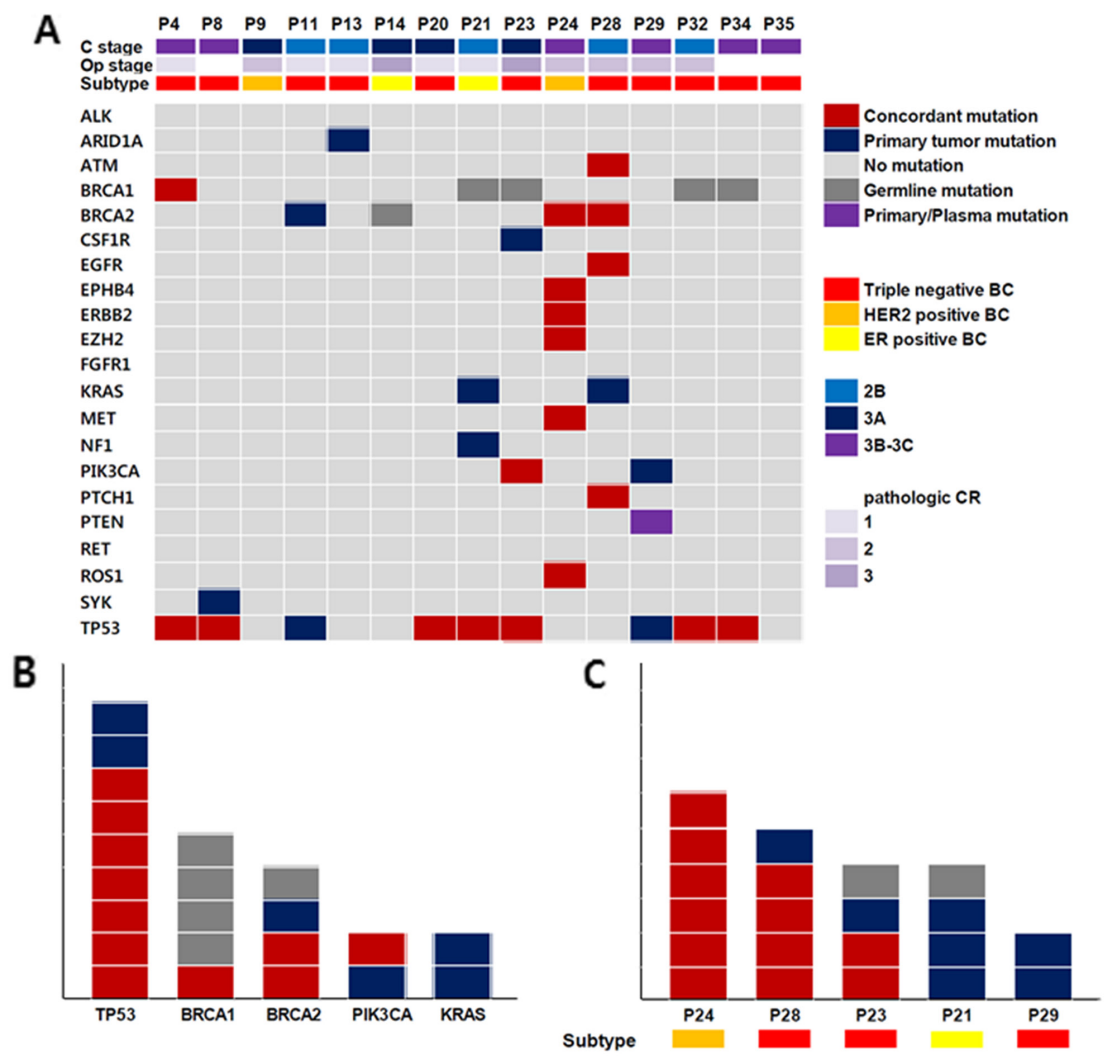
**(A)** Clinicopathologic characteristics and genetic aberrations across 15 breast cancer **(B, C)**, concordant mutation (m): same SNVs detection in ctDNA and BC tissue, primary tumor m: SNV detection in BC tissue, Germline m: same SNVs detection in ctDNA, BC tissue and white blood cells, Primary/plasma mutation: SNV detection in same gene but not same locus, (B) Genes with frequent SNV detection, (C) Samples with frequent SNV detection.

The *TP53* mutation was most commonly detected in primary tumor tissues and plasma (Figure 1). In both tumor and plasma ctDNA, 7 (46.7%) concordant *TP53* mutations were detected and 2 (13.3%) in primary tumors only. *BRCA1* and *BRCA2* mutations were also frequently detected. Somatic *BRCA1* and *BRCA2* mutations were detected in 4 BCs (1 *BRCA1* and 3 *BRCA2*) and germline mutations in 5 (4 *BRCA1* and 1 *BRCA2*). Of the somatic *BRCA1* mutations, one was detected only in plasma DNA and not in the primary tumor.

### Serial change of SNVs in plasma and tumor response to neoadjuvant chemotherapy

Serial plasma DNAs were collected at BC diagnosis, after the 1^st^ NAC cycle, BC surgery and 6 months after BC surgery to analyze the association between a quantitative shift of SNVs in plasma and a tumor response to NAC. For this analysis, only plasma SNVs that were detected in primary tumors were used.

At BC diagnosis, we analyzed the relationship between plasma SNV quantity and clinical stage. In this analysis, the amount of plasma SNVs did not correlate with clinical stage at diagnosis (Table 1 and Supplementary Table 3).

**Figure 2:**
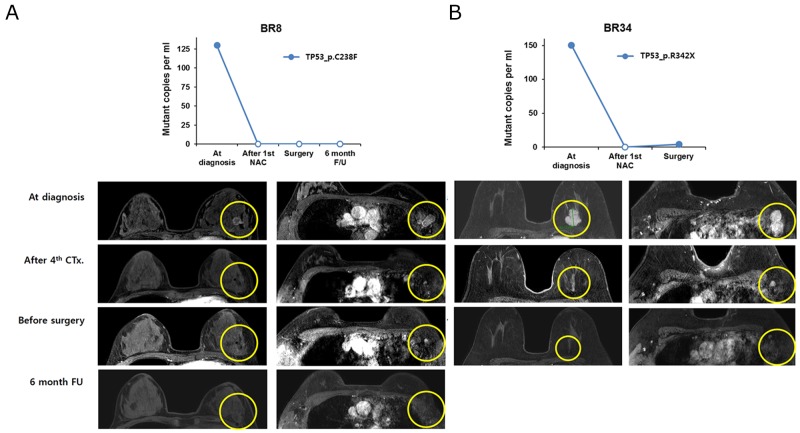
The change in ctDNA amount and MRI imaging in patients with pathologic complete remission after neoadjuvant chemotherapy **(A)** BR8 and **(B)** BR34 patients.

For the therapeutic effects of NAC, we found that ctDNA disappeared after the 1^st^ cycle of NAC in two samples that achieved pCR (BR8, BR34) (Figure 2). In BR8, no ctDNA was observed from the 1^st^ NAC cycle to 6 months after surgery. Although minute amounts of ctDNA (4 copies/ml) were detected in the BR34 plasma sample obtained prior to surgery, ctDNA estimated to be 151 copies/ml at diagnosis was not detectable after the 1^st^ NAC. In contrast, tumors with ctDNA after the 1st NAC cycle did not achieve pCR.

**Figure 3:**
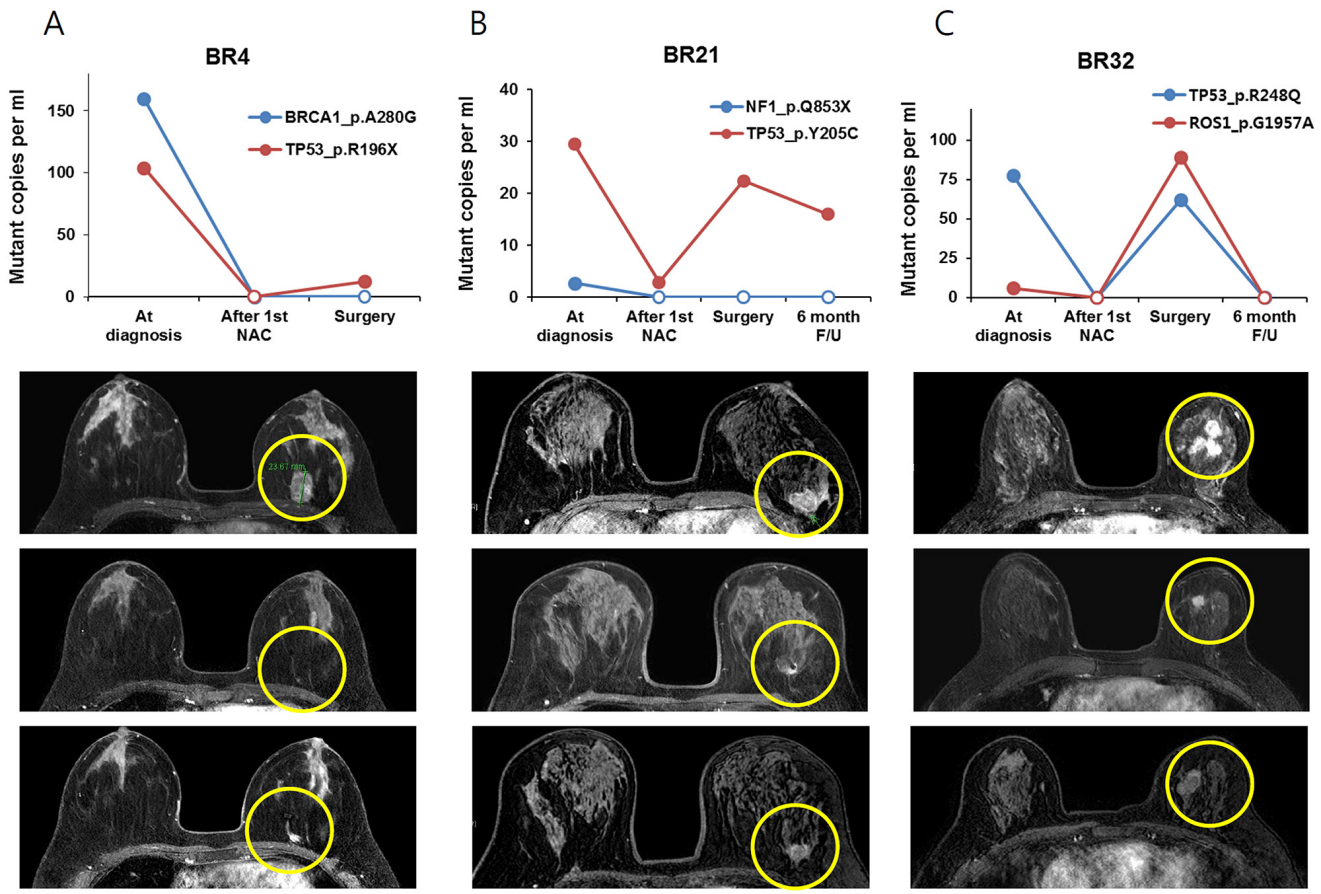
The change of ctDNA amount and MRI imaging in patients with residual tumor after NAC, tumor size decreased after the first 4cycles of NAC and then tumor size increased during the last 4cycles of NAC **(A)** BR4 **(B)** BR21 and **(C)** BR32 patients.

In addition, we did not find an association between the amount of ctDNA at curative surgery and residual cancer burden. BR23 and 24 had a high burden of ctDNA at operation and a high score of residual cancer burden, but BR32 had high residual cancer burden scores with a relatively low amount of ctDNA (Table 1 and Figures 2 and 3).

**Figure 4:**
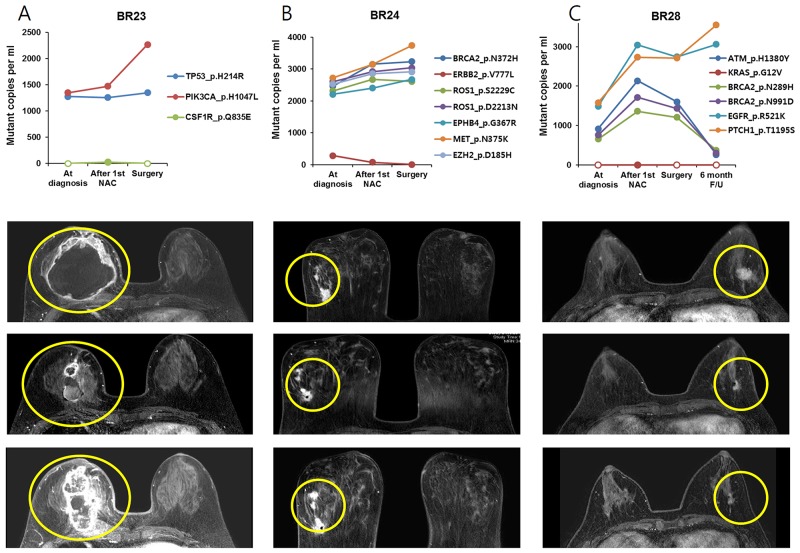
The change of ctDNA amount and MRI imaging in patients with residual tumor after NAC, tumor did not respond during NAC in patients **(A)** BR23, **(B)** BR24 and **(C)** BR28.

The relationship between plasma SNV variation and tumor response detected by breast MRI was also analyzed (Figure 3). The amount of ctDNA decreased after the 1st NAC cycle and increased at the time of surgery in three BCs (BR4, BR21 and BR32). Serial breast MRI showed tumor shrinkage after the first cycle of NAC but an increase after full cycles of NAC in the three BCs. Another three BCs (BR23, BR24 and BR28) had minimal response to NAC and ctDNA did not decrease (Figure 4).

### Association between amount of plasma SNVs and allele frequency in tumor biopsy

**Figure 5:**
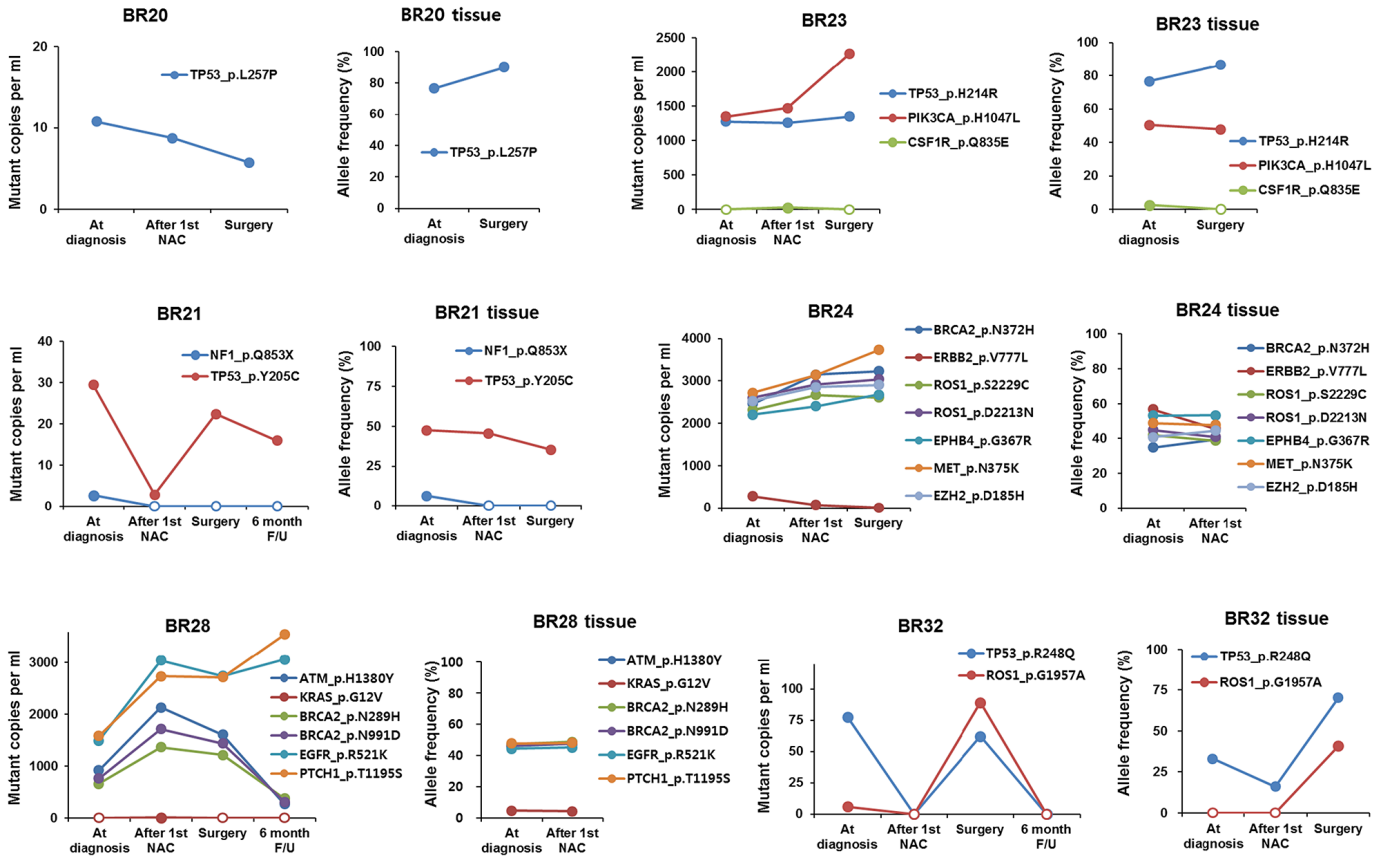
The association between ctDNA amount and allele frequency in tumor biopsy

Collecting sequential biopsies from seven BCs in parallel with blood draws, we detected and compared SNVs from both plasma and tumor biopsy samples. While the allele frequency for a given SNV in tumor tissue samples rarely predicted its copy number or allele frequency in cell-free DNA, we found that the relative frequencies between different SNVs correlated between paired plasma and tissue biopsy samples to a certain degree (Figure 5). Because the ratio of copy numbers between two SNVs mirrored that of allele frequencies in plasma samples, we assumed the relative copy numbers between SNVs were a direct indicator of the relative allele frequencies between SNVs. We found *TP53* p.Y205C at dramatically higher frequencies than *NF1* p.Q853X consistently in both plasma and tissue biopsy samples from BR21. In BR32, *TP53* p.R248Q was predominantly detected at the time of diagnosis and the frequency of *ROS1* p.G1957A became comparable to that of *TP53* p.R248Q at surgery. This change was also observed in both plasma and tissue biopsy samples, which suggested the expansion of a subpopulation harboring the two mutations.

On the other hand, there were also discrepancies between plasma and tumor tissue specimens, probably because subpopulations with unique variants released ctDNA at variable rates. In BR24, for example, seven variants detected in tissue biopsies displayed a frequency in a range between 35-55%, which did not change much after the 1^st^ cycle of NAC. In plasma, however, *ERBB2* p.V777L was detected at a dramatically lower frequency than the other six variants. As another example, ctDNA analysis in BR28 showed that *EGFR* p.R521K and *PTCH1* p.T1195S variants were more frequent than *ATM* pH1380Y, *BRCA2* p.N289H, and *BRCA2* p.N991D, whereas all these variants were present at a similar frequency in tumor tissue samples at diagnosis and after the 1^st^ NAC cycle. These data indicated the presence of distinct subpopulations in the example cases. Thus, our results suggested that comparison of relative SNV allele frequencies between plasma and tumor biopsy samples enable us to better understand intra-tumor heterogeneity by uncovering subclonality unidentifiable in tissue biopsy or plasma data alone.

## DISCUSSION

We performed targeted deep sequencing of both plasma SNVs and tumor biopsies. In this analysis, plasma SNVs after the 1^st^ NAC cycle represented tumor response to 8 cycles of NAC and variation in plasma SNVs was also associated with tumor response. In contrast, the absolute value of plasma SNVs did not correlate with tumor burden.

The detection of circulating tumor DNA requires the technique of somatic mutation identification in cancer patients. The technique of ctDNA detection has continuously advanced. A PCR-based method has been superseded by next generation sequencing-based methods to detect ctDNA. PCR-based methods such as bead-based digital PCR in emulsions (BEAMing) and droplet-based digital PCR have been used to detect highly recurrent tumor-specific mutations in well-known driver genes such as *APC, BRAF, KRAS* and *EGFR* in plasma samples [15-19]. However, most patients do not benefit from these methods due to a lack of mutations in these genes. Moreover, identification of mutations in tumor suppressor genes such as *TP53*, which are mutated in a variety of cancers but lack well-defined hotspot mutations, is challenging using these methods. As targeted deep sequencing became widespread for profiling genetic alterations in primary tumor tissues [20], sequencing-based methods have also been used to detect ctDNA as a more suitable alternative for a range of genomic regions [14, 21, 22].

Most studies of ctDNA in breast cancer focus on monitoring hotspots on single or several genes [12, 13]. These studies use hotspot mutation of unique genes in primary BC biopsy tissue to monitor somatic mutations. Accordingly, only one somatic mutation was used to monitor BC status. In this study, we tested multiple hotspot mutations of multiple genes in primary biopsy and monitored multiple SNVs in plasma DNA. Because this panel is not a BC specific gene panel, some genes known to be associated with BC, such as *ESR1*, *RB1* and *CCND1* were not included. Nevertheless, subclonal response to NAC could be observed and chemoresistant clones were more precisely found through ctDNA monitoring using this multi-gene panel. This information is helpful to treat BC patients without pCR after NAC, who are expected to have a short disease-free duration, by individualizing adjuvant treatment according to resistant subclones.

Currently, most BC patients receive 6–8 cycles of NAC. During 4–6 months of NAC, many patients suffer severe adverse events and some patients do not achieve sufficient benefit from NAC. In this study, we hypothesized that ctDNA monitoring only after the 1^st^ NAC cycle would predict tumor response after entire cycles of NAC. Moreover, based on chemoresistant subclones, personalized NAC may result in increased efficacy and decreased toxicity might be possible.

We also compared relative SNV allele frequencies between plasma and BC tissue to reveal intra-tumor heterogeneity. In BR28, for example, relatively high frequencies of *EGFR* p.R521K and *PTCH1* p.T1195S over *ATM* p.H1380Y, *BRCA2* p.N289H, and *BRCA2* p.N991D were observed in plasma compared to tumor tissue data, indicating tumor heterogeneity. Furthermore, by monitoring the relative levels of SNVs in sequential plasma samples over time, we were able to capture molecular changes during tumor clonal evolution. In sequential plasma samples from BR28, we found that *EGFR* p.R521K and *PTCH1* p.T1195S variants increased 6 months after surgery, whereas *ATM* p.H1380Y, *BRCA2* p.N289H, and *BRCA2* p.N991D variants decreased at that time. This data implied expansion of a subpopulation with *EGFR* p.R521K and *PTCH1* p.T1195S, which also supported the presence of subpopulations identified by comparison of relative variant copy numbers in plasma with relative variant frequencies in tumor tissue data. In addition, BR24 and BR 32 indicated the expansion or decline of a particular subpopulation with unique mutations based on changes in relative plasma SNV levels.

Therefore, our results suggest that longitudinal tracking of ctDNA should indicate tumor biology more precisely compared to tumor biopsy, by providing information on tumor intercellular heterogeneity and/or clonal evolution.

CtDNA originates from dead tumor cells and is highly fragmented. Therefore, the detection rate for ctDNA is around 60–70% in early breast cancer patients [13]. In our study, the detection rate was 86.7% and all BCs with somatic alterations in primary tumors had plasma SNVs. However, only targeted deep sequencing was performed and might not have detected some SNVs and some BC associated genes were not included. Whole-genome sequencing of plasma and tumor tissues is warranted to more precisely monitor plasma SNVs, although cost and time may be obstacles. Short follow-up duration was a limitation in this study and we did not evaluate disease recurrence, but only residual cancer burden at surgery. Currently, patients in this study are undergoing regular follow-up and further survival analysis is warranted.

This study is the first attempt to apply a targeted ultra-deep sequencing method to evaluate the level of ct-DNA across 82 cancer-related genes during NAC, overcoming the limitations of methods targeting only a small number of predefined mutations. This targeted ultra-deep sequencing for ctDNA analysis would be a clinically useful method to monitor not only tumor burden but also drug resistance. Most of all, we suggest that ctDNA could be the earliest predictor of NAC response. Further large-scaled study for precision treatment based on ctDNA analysis is warranted.

## MATERIALS AND METHODS

### Patients

Twenty patients set to receive NAC were enrolled. This prospective study of BC genomics was approved by the Institutional Review Board of Samsung Medical Center, Seoul, Korea (2014-11-015). Written informed consent was obtained from all participants. Patients were diagnosed with LABC using breast magnetic resonance imaging (MRI), chest/abdomen-pelvis computed tomography (CT) and bone scan, and were treated with sequential anthracycline-taxane-based chemotherapy. Trastuzumab was allowed depending on HER2 status. All patients received curative surgery after NAC, followed by adjuvant radiotherapy, except for patients who had disease progression. Serial BC core biopsies and blood samplings were taken at baseline and after the 1st of cycle NAC. After NAC, surgical specimens were obtained from curative BC surgery; blood sampling was also performed at this time (NCT02591966).

### Plasma and PBL sample collection

Blood samples were drawn into Cell-Free DNA™ BCT tubes (Streck Inc., Omaha, NE, USA) [23] and processed within 6 h of collection by differential centrifugal sedimentation (840 x *g* for 10 min, 1040 x *g* for 10 min, and 5000 x *g* for 10 min at room temperature). Peripheral blood leukocytes (PBLs) were collected from the initial centrifugation. Plasma and PBL samples were stored at −80 Celsius until cfDNA extraction.

### DNA extraction

Germline genomic DNA from PBLs was purified by QIAamp DNA mini kits (Qiagen, Santa Clarita, CA, USA). Circulating cfDNA was extracted from 2 to 5 mL plasma using QIAamp Circulating Nucleic Acid kits (Qiagen). DNA concentration and purity were assessed by Nanodrop 8000 UV-Vis spectrometer (Thermo Fisher Scientific, Waltham, MA, USA) and a Qubit 2.0 fluorometer using Picogreen fluorescence assays (Life Technologies, Grand Island, NY, USA). Fragment size distribution was estimated using a 2200 TapeStation Instrument (Agilent Technologies, Santa Clara, CA, USA) and real-time PCR Mx3005p (Agilent Technologies) according to the manufacturer’s manual.

### Hybrid capture-based targeted sequencing

Genomic DNA from PBL and primary tissue specimens was acoustically sheared to 150-200 bp using a Covaris S2 (7 min, 0.5% duty, intensity = 0.1, 50 cycles/burst; Covaris Inc. Woburn, MA, USA). Plasma DNA was used for library construction without fragmentation. The sequencing libraries for primary tumor tissue samples were created by using SureSelect XT reagent kit, HSQ (Agilent Technologies) following the manufacturer’s recommended protocols. Sequencing libraries for PBL and plasma DNAs were constructed with KAPA Hyper Prep kits (Kapa Biosystems, Woburn, MA, USA) [24]. DNA fragments were ligated with pre-indexed PentAdapter™ (PentaBase ApS, Denmark) at 4°C overnight, purified using AMPure XP beads (Beckman Coulter, Indianapolis, IN, USA), amplified with P5 and P7 oligonucleotides, and subjected to hybrid selection for target enrichment. For hybrid selection, we designed unique RNA baits that targeted ∼202 kb of the human genome, including exons from 82 cancer-related genes (Supplementary Table 1). Multiplex hybrid selections pooling up to eight libraries were carried out following the SureSelect bait hybridization protocol with IDT xGen blocking oligonucleotide (IDT, Santa Clara, CA, USA) for the pre-indexed adapters. After the target enrichment step, captured DNA fragments were amplified and purified. Libraries were normalized to an equal concentration of 2 nM and pooled by equal volume. After denaturing the pooled libraries, cluster amplification was performed according to the manufacturer’s protocol (Illumina, San Diego, CA, USA). Flow cells were sequenced in 100-bp paired-end mode using HiSeq 2500 v3 Sequencing-by-Synthesis Kits (Illumina) and analyzed using RTA v.1.12.4.2 or later.

### Sequence data processing

Using BWA-mem (v 0.7.5) [25], all raw data were aligned to the hg19 human reference, creating BAM files. SAMTOOLS (v 0.1.18) [26] were used for sorting SAM/BAM files, Picard (v 1.93), for local realignment, and GATK (v 3.1.1) [27] for duplicate markings. Through this process, we filtered reads to remove duplicates, discordant pairs, and off-target reads. Quality control (QC) was assessed using a custom Perl script to collect various sequencing metrics such as read alignment rate, duplicate rate, and on-target rate.

### SNV detection in primary tissues and statistical test for SNV presence in plasma

MuTect 1.1.4 [28] and Varscan2 [29] were employed to detect somatic SNVs in primary tumor tissues with matched germline samples. Default parameter values were used with some modifications for Varscan2 as previously described [14]. Somatic SNVs in tumor tissues called by at least one of the methods were retained if they were present at a frequency greater than 2% and supported by more than 10 unique reads. Germline variants were filtered out if they were present at a frequency greater than 0.5% in the matched PBL sample. Somatic SNVs detected in primary tumor tissues were listed and tested for presence in paired plasma samples as described previously [14]. To mitigate the impact of sequencing errors on the variant detection in plasma DNA, we considered only high quality (Phred quality scores ≥30) bases by filter out low quality bases during mpileup run. The allelic fraction for individual non-reference alleles in each sample was adjusted by position-specific error rates in order to minimize the influence of background error level. Then, allele frequencies of a given SNV were tested to see if they ranked in the 95^th^ percentile of adjusted frequencies of background alleles. The average position-specific error rates across the entire target regions were calculated from 55 plasma DNA samples. The overall mean background allele frequency was estimated to be 0.007% and 0.008% in plasma and PBL DNA samples.

### Biopsy-free SNV identification in plasma DNA

A detection method modified from previous studies [14, 30] was established to identify candidate tumor-derived SNVs in plasma DNA. First, positions with strand bias >0.9 and total read depth <500× were filtered out. After filtering out germline variants (AF >0.5% in matched PBL sample), a binomial test was performed to examine if a non-reference allele was significantly more abundant in plasma DNA than matched germline DNA. Multiple testing corrections were made by Bonferroni adjustment with a significance level of 0.05. Next, we performed Z-tests to examine if the filtered non-reference alleles were present at a significantly elevated level in the test sample compared to other plasma DNA samples [14]. For comparison, a background allele frequency distribution was generated by selecting non-reference alleles of plasma samples (n = 53) present at a frequency <2.5% in the paired tumor and <0.5% in the paired germline DNA, and displaying a sufficient total depth at their positions in all matched samples (>250× in primary tumor tissue, >500× in PBL, and >500× in plasma DNA). The following filters were applied: (1) candidate alleles with less than seven supporting reads were discarded; (2) when two or more candidates were within any 10-bp window, all with allele frequency less than 20% were discarded [30]; and (3) candidates with the Bonferroni adjusted p-value higher than 10^-18^ from the Z-test were discarded. Nonsynonymous, stop-gain, and splice-disrupting SNVs were included to list the final positive calls.

## SUPPLEMENTARY MATERIALS FIGURE AND TABLES





## References

[1] Jordan NV, Bardia A, Wittner BS, Benes C, Ligorio M, Zheng Y, Yu M, Sundaresan TK, Licausi JA, Desai R, O’Keefe RM, Ebright RY, Boukhali M (2016). HER2 expression identifies dynamic functional states within circulating breast cancer cells. Nature.

[2] Bonadonna G (1992). Evolving concepts in the systemic adjuvant treatment of breast cancer. Cancer Res.

[3] von Minckwitz G, Untch M, Blohmer JU, Costa SD, Eidtmann H, Fasching PA, Gerber B, Eiermann W, Hilfrich J, Huober J, Jackisch C, Kaufmann M, Konecny GE (2012). Definition and impact of pathologic complete response on prognosis after neoadjuvant chemotherapy in various intrinsic breast cancer subtypes. J Clin Oncol.

[4] Symmans WF, Peintinger F, Hatzis C, Rajan R, Kuerer H, Valero V, Assad L, Poniecka A, Hennessy B, Green M, Buzdar AU, Singletary SE, Hortobagyi GN (2007). Measurement of residual breast cancer burden to predict survival after neoadjuvant chemotherapy. J Clin Oncol.

[5] Hannemann J, Oosterkamp HM, Bosch CA, Velds A, Wessels LF, Loo C, Rutgers EJ, Rodenhuis S, van de Vijver MJ (2005). Changes in gene expression associated with response to neoadjuvant chemotherapy in breast cancer. J Clin Oncol.

[6] Symmans WF, Volm MD, Shapiro RL, Perkins AB, Kim AY, Demaria S, Yee HT, McMullen H, Oratz R, Klein P, Formenti SC, Muggia F (2000). Paclitaxel-induced apoptosis and mitotic arrest assessed by serial fine-needle aspiration: implications for early prediction of breast cancer response to neoadjuvant treatment. Clin Cancer Res.

[7] Jones RL, Salter J, A’Hern R, Nerurkar A, Parton M, Reis-Filho JS, Smith IE, Dowsett M (2009). The prognostic significance of Ki67 before and after neoadjuvant chemotherapy in breast cancer. Breast Cancer Res Treat.

[8] Denkert C, Loibl S, Noske A, Roller M, Muller BM, Komor M, Budczies J, Darb-Esfahani S, Kronenwett R, Hanusch C, von Torne C, Weichert W, Engels K (2010). Tumor-associated lymphocytes as an independent predictor of response to neoadjuvant chemotherapy in breast cancer. J Clin Oncol.

[9] Esserman LJ, Berry DA, Cheang MC, Yau C, Perou CM, Carey L, DeMichele A, Gray JW, Conway-Dorsey K, Lenburg ME, Buxton MB, Davis SE, van’t Veer LJ (2012). Chemotherapy response and recurrence-free survival in neoadjuvant breast cancer depends on biomarker profiles: results from the I-SPY 1 TRIAL (CALGB 150007/150012; ACRIN 6657). Breast Cancer Res Treat.

[10] Crowley E, Di Nicolantonio F, Loupakis F, Bardelli A (2013). Liquid biopsy: monitoring cancer-genetics in the blood. Nat Rev Clin Oncol.

[11] Diaz LA, Bardelli A (2014). Liquid biopsies: genotyping circulating tumor DNA. J Clin Oncol.

[12] Dawson SJ, Tsui DW, Murtaza M, Biggs H, Rueda OM, Chin SF, Dunning MJ, Gale D, Forshew T, Mahler-Araujo B, Rajan S, Humphray S, Becq J (2013). Analysis of circulating tumor DNA to monitor metastatic breast cancer. N Engl J Med.

[13] Garcia-Murillas I, Schiavon G, Weigelt B, Ng C, Hrebien S, Cutts RJ, Cheang M, Osin P, Nerurkar A, Kozarewa I, Garrido JA, Dowsett M, Reis-Filho JS (2015). Mutation tracking in circulating tumor DNA predicts relapse in early breast cancer. Sci Transl Med.

[14] Newman AM, Bratman SV, To J, Wynne JF, Eclov NC, Modlin LA, Liu CL, Neal JW, Wakelee HA, Merritt RE, Shrager JB, Loo BW, Alizadeh AA (2014). An ultrasensitive method for quantitating circulating tumor DNA with broad patient coverage. Nat Med.

[15] Diehl F, Li M, Dressman D, He Y, Shen D, Szabo S, Diaz LA, Goodman SN, David KA, Juhl H, Kinzler KW, Vogelstein B (2005). Detection and quantification of mutations in the plasma of patients with colorectal tumors. Proc Natl Acad Sci U S A.

[16] Diaz LA, Williams RT, Wu J, Kinde I, Hecht JR, Berlin J, Allen B, Bozic I, Reiter JG, Nowak MA, Kinzler KW, Oliner KS, Vogelstein B (2012). The molecular evolution of acquired resistance to targeted EGFR blockade in colorectal cancers. Nature.

[17] Misale S, Yaeger R, Hobor S, Scala E, Janakiraman M, Liska D, Valtorta E, Schiavo R, Buscarino M, Siravegna G, Bencardino K, Cercek A, Chen CT (2012). Emergence of KRAS mutations and acquired resistance to anti-EGFR therapy in colorectal cancer. Nature.

[18] Oxnard GR, Paweletz CP, Kuang Y, Mach SL, O’Connell A, Messineo MM, Luke JJ, Butaney M, Kirschmeier P, Jackman DM, Janne PA (2014). Noninvasive detection of response and resistance in EGFR-mutant lung cancer using quantitative next-generation genotyping of cell-free plasma DNA. Clin Cancer Res.

[19] Douillard JY, Ostoros G, Cobo M, Ciuleanu T, Cole R, McWalter G, Walker J, Dearden S, Webster A, Milenkova T, McCormack R (2014). Gefitinib treatment in EGFR mutated caucasian NSCLC: circulating-free tumor DNA as a surrogate for determination of EGFR status. J Thorac Oncol.

[20] Frampton GM, Fichtenholtz A, Otto GA, Wang K, Downing SR, He J, Schnall-Levin M, White J, Sanford EM, An P, Sun J, Juhn F, Brennan K (2013). Development and validation of a clinical cancer genomic profiling test based on massively parallel DNA sequencing. Nat Biotechnol.

[21] Forshew T, Murtaza M, Parkinson C, Gale D, Tsui DW, Kaper F, Dawson SJ, Piskorz AM, Jimenez-Linan M, Bentley D, Hadfield J, May AP, Caldas C (2012). Noninvasive identification and monitoring of cancer mutations by targeted deep sequencing of plasma DNA. Sci Transl Med.

[22] Bettegowda C, Sausen M, Leary RJ, Kinde I, Wang Y, Agrawal N, Bartlett BR, Wang H, Luber B, Alani RM, Antonarakis ES, Azad NS, Bardelli A (2014). Detection of circulating tumor DNA in early- and late-stage human malignancies. Sci Transl Med.

[23] Norton SE, Lechner JM, Williams T, Fernando MR (2013). A stabilizing reagent prevents cell-free DNA contamination by cellular DNA in plasma during blood sample storage and shipping as determined by digital PCR. Clin Biochem.

[24] Chung J, Son DS, Jeon HJ, Kim KM, Park G, Ryu GH, Park WY, Park D (2016). The minimal amount of starting DNA for Agilent’s hybrid capture-based targeted massively parallel sequencing. Sci Rep.

[25] Li H, Durbin R (2010). Fast and accurate long-read alignment with Burrows-Wheeler transform. Bioinformatics.

[26] Li H, Handsaker B, Wysoker A, Fennell T, Ruan J, Homer N, Marth G, Abecasis G, Durbin R (2009). The Sequence Alignment/Map format and SAMtools. Bioinformatics.

[27] McKenna A, Hanna M, Banks E, Sivachenko A, Cibulskis K, Kernytsky A, Garimella K, Altshuler D, Gabriel S, Daly M, DePristo MA (2010). The Genome Analysis Toolkit: a MapReduce framework for analyzing next-generation DNA sequencing data. Genome Res.

[28] Cibulskis K, Lawrence MS, Carter SL, Sivachenko A, Jaffe D, Sougnez C, Gabriel S, Meyerson M, Lander ES, Getz G (2013). Sensitive detection of somatic point mutations in impure and heterogeneous cancer samples. Nat Biotechnol.

[29] Koboldt DC, Zhang Q, Larson DE, Shen D, McLellan MD, Lin L, Miller CA, Mardis ER, Ding L, Wilson RK (2012). VarScan 2: somatic mutation and copy number alteration discovery in cancer by exome sequencing. Genome Res.

[30] Takai E, Totoki Y, Nakamura H, Morizane C, Nara S, Hama N, Suzuki M, Furukawa E, Kato M, Hayashi H, Kohno T, Ueno H, Shimada K (2015). Clinical utility of circulating tumor DNA for molecular assessment in pancreatic cancer. Sci Rep.

