# Supplementary material for: Circulating tumor DNA shows variable clonal response of breast cancer during neoadjuvant chemotherapy
Source: Oncotarget. 2017 Sep 23;8(49):86423–34. doi: 10.18632/oncotarget.21198 (PMC5689695; doi:10.18632/oncotarget.21198)
Supplement: Supplementary file 1 [file oncotarget-08-86423-s001.pdf]

Circulating tumor DNA shows variable clonal response of breast cancer during neoadjuvant chemotherapy

SUPPLEMENTARY MATERIALS

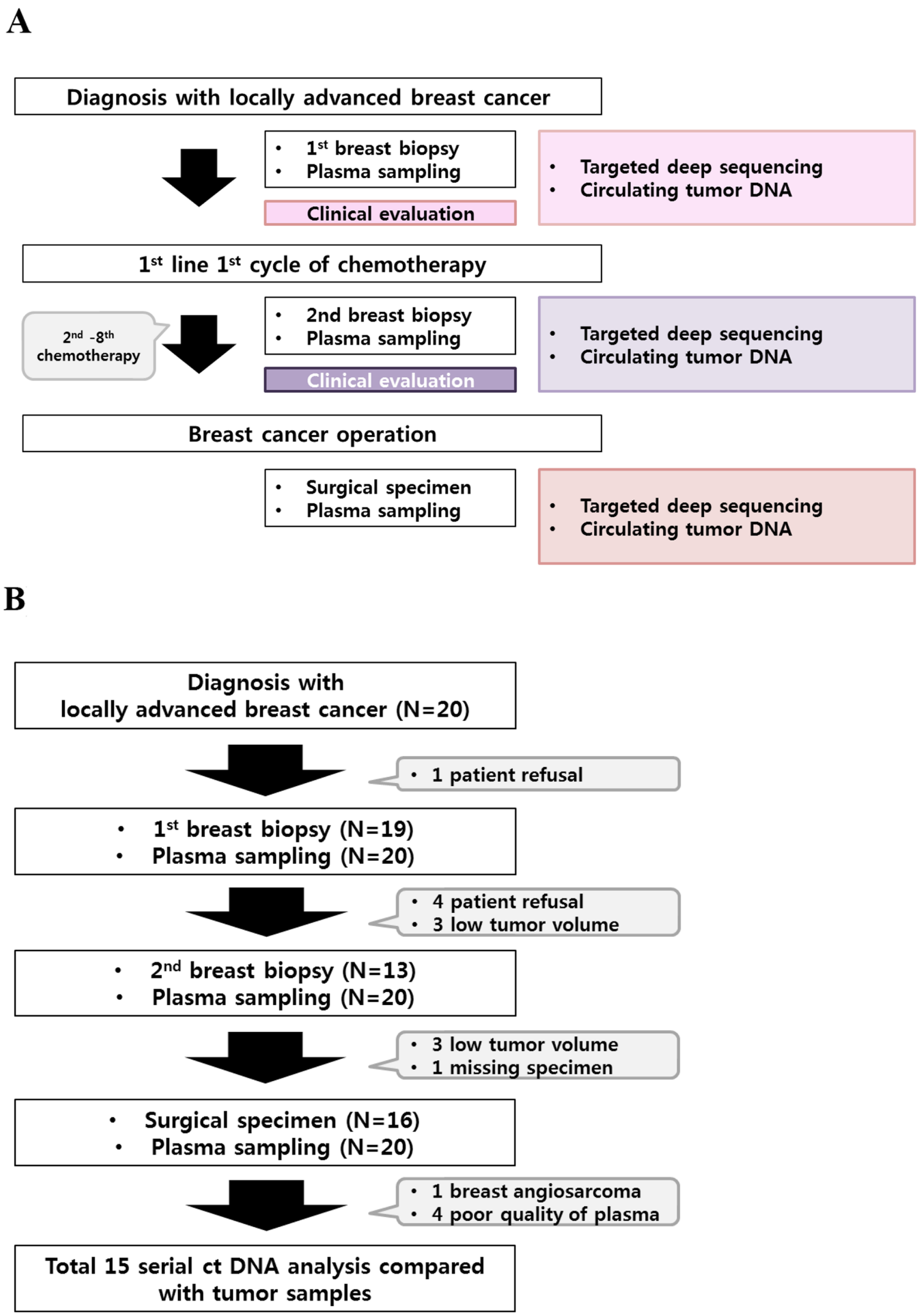

Supplementary Figure 1: (A) Concept of the experiment **(B)** Flowchart for experiment.

Supplementary Table 1: 82 cancer gene list

ABL1	ARID2	BRCA2	EGFR	FGFR1	HRAS	KDR	NF1	PIK3R1	SMAD4
AKT1	ATM	CDH1	EPHB4	FGFR2	IDH1	KIT	NOTCH1	PTCH1	SMARCB1
AKT2	ATRX	CDK4	ERBB2	FGFR3	IDH2	KRAS	NPM1	PTCH2	SMO
AKT3	AURKA	CDK6	ERBB3	FLT3	IGF1R	MDM2	NRAS	PTEN	SRC
ALK	AURKB	CDKN2A	ERBB4	GNA11	ITK	MET	NTRK1	PTPN11	STK11
APC	BCL2	CSF1R	EWSR1	GNAQ	JAK1	MLH1	PDGFRA	RB1	SYK
ARID1A	BRAF	CTNNB1	EZH2	GNAS	JAK2	MPL	PDGFRB	RET	TMPRSS2
ARID1B	BRCA1	DDR2	FBXW7	HNF1A	JAK3	MTOR	PIK3CA	ROS1	TOP1
								TP53	VHL

Supplementary Table 2: Summary of sequencing metrics

		Total read	No. of reads mapped	Duplication rate	On target base	On target rate	Median depth
Plasma	Mean	35773700	1283872820	63.7	470489437	44.1	2289
	SD	16848265	657976988	10.4	273929362	10.0	1326
WBC	Mean	38940588	2764860335	29.5	996635915	48.2	2404
	SD	7898747	863840070.5	15.6	416306950	8.8	1711
Primary	Mean	36946511	2871059317	19.6	1079375820	42.6	1187
	SD	11059113	1168009865	13.3	443535313	14.6	659

Supplementary Table 3: Summary of circulating cell-free DNA preparation.

See Supplementary File 1
