# Supplementary material for: Circulating tumor DNA shows variable clonal response of breast cancer during neoadjuvant chemotherapy
Source: Oncotarget. 2017 Sep 23;8(49):86423–34. doi: 10.18632/oncotarget.21198 (PMC5689695; doi:10.18632/oncotarget.21198)
Supplement: Supplementary file 2 [file oncotarget-08-86423-s002.docx]

**Supplementary Table 3: Summary of circulating cell-free DNA preparation.**

| **PlasmaID** | **Mean depth** | **plasma(ml)** | **qubit(ng/ul)** | **total cfDNA(ng)** | **used in seq(ng)** | **used in seq(ul)** |
| --- | --- | --- | --- | --- | --- | --- |
| BR4.1 | 2661.187 | 3.8 | 0.699 | 48.93 | 34.95 | 50 |
| BR4.2 | 3044.85 | 3.8 | 1.27 | 88.9 | 63.5 | 50 |
| BR4.3 | 4299.007 | 5 | 3.1 | 217 | 155 | 50 |
| BR7.1 | 2210.924 | 3 | 1.07 | 74.9 | 53.5 | 50 |
| BR7.2 | 4611.252 | 5 | 3.74 | 261.8 | 187 | 50 |
| BR7.3 | 1624.045 | 4 | 1.67 | 116.9 | 83.5 | 50 |
| BR8.1 | 1652.047 | 3 | 0.552 | 38.64 | 27.6 | 50 |
| BR8.2 | 1723.121 | 3 | 0.83 | 58.1 | 41.5 | 50 |
| BR8.3 | 2759.918 | 4 | 1.73 | 121.1 | 86.5 | 50 |
| BR8.4 | 2774.428 | 4.5 | 0.82 | 57.4 | 40 | 49 |
| BR9.1 | 2333.914 | 4 | 0.8 | 56 | 40 | 50 |
| BR9.2 | 2261.166 | 3.5 | 1.33 | 93.1 | 66.5 | 50 |
| BR9.3 | 2122.219 | 4 | 1.06 | 74.2 | 53 | 50 |
| BR11.1 | 1068.891 | 4 | 0.41 | 28.7 | 20.5 | 50 |
| BR11.2 | 2104.588 | 5 | 0.84 | 58.8 | 42 | 50 |
| BR11.3 | 1999.227 | 5 | 0.74 | 51.8 | 37 | 50 |
| BR11.4 | 110.6642 | 4 | 6 | 420 | 54 | 9 |
| BR13.1 | 850.9298 | 3.3 | 0.36 | 25.2 | 18 | 50 |
| BR13.2 | 695.9022 | 4.5 | 0.37 | 25.9 | 18.5 | 50 |
| BR13.3 | 1234.798 | 4.5 | 0.47 | 32.9 | 23.5 | 50 |
| BR13.4 | 900.1033 | 4 | 0.82 | 57.4 | 40 | 50 |
| BR14.1 | 3084.252 | 5 | 1.45 | 101.5 | 72.5 | 50 |
| BR14.2 | 2649.293 | 5 | 1.32 | 92.4 | 66 | 50 |
| BR14.3 | 2818.486 | 5 | 1.31 | 91.7 | 65.5 | 50 |
| BR20.1 | 1934.964 | 3 | 0.736 | 51.52 | 36.8 | 50 |
| BR20.2 | 1883.432 | 2.5 | 0.646 | 45.22 | 32.3 | 50 |
| BR20.3 | 3649.052 | 4 | 1.98 | 138.6 | 99 | 50 |
| BR21.1 | 2001.357 | 3 | 0.758 | 53.06 | 37.9 | 50 |
| BR21.2 | 3157.982 | 4 | 1.67 | 116.9 | 83.5 | 50 |
| BR21.3 | 3949.486 | 4 | 2.38 | 166.6 | 100 | 42 |
| BR21.4 | 1363.974 | 4 | 1.37 | 95.9 | 51 | 37 |
| BR23.1 | 3185.827 | 4.7 | 1.67 | 116.9 | 83.5 | 50 |
| BR23.2 | 2426.083 | 4 | 6.54 | 457.8 | 100 | 35 |
| BR23.3 | 3584.078 | 4 | 3.88 | 271.6 | 100 | 24 |
| BR24.1 | 1501.981 | 3.5 | 0.928 | 64.96 | 46.4 | 50 |
| BR24.2 | 3065.035 | 4.5 | 1.31 | 91.7 | 65.5 | 50 |
| BR24.3 | 2629.079 | 4 | 1.32 | 92.4 | 66 | 50 |
| BR28.1 | 1137.947 | 4 | 0.41 | 28.7 | 20.5 | 50 |
| BR28.2 | 2097.324 | 4.7 | 0.85 | 59.5 | 42.5 | 50 |
| BR28.3 | 2020.344 | 4.7 | 0.76 | 53.2 | 38 | 50 |
| BR28.4 | 1445.28 | 4 | 2.28 | 159.6 | 50 | 22 |
| BR29.1 | 1445.28 | 2.5 | 1.58 | 110.6 | 79 | 50 |
| BR29.2 | 1422.966 | 3.2 | 0.721 | 50.47 | 36.05 | 50 |
| BR29.3 | 3133.603 | 3.7 | 1.37 | 95.9 | 68.5 | 50 |
| BR32.1 | 727.5579 | 4 | 0.41 | 28.7 | 20.5 | 50 |
| BR32.2 | 1533.019 | 3.5 | 0.42 | 29.4 | 21 | 50 |
| BR32.3 | 3747.685 | 4 | 1 | 70 | 50 | 50 |
| BR32.4 | 665.1663 | 4 | 0.8 | 56 | 39 | 49 |
| BR34.1 | 1901.007 | 4 | 1.02 | 71.4 | 51 | 50 |
| BR34.2 | 1274.325 | 3.8 | 0.53 | 37.1 | 26.5 | 50 |
| BR34.3 | 2798.478 | 3.7 | 1.02 | 71.4 | 51 | 50 |
| BR35.1 | 1765.792 | 4.5 | 0.64 | 44.8 | 32 | 50 |
| BR35.2 | 911.6882 | 4 | 1.67 | 116.9 | 83.5 | 50 |
| BR35.3 | 1039.709 | 4 | 0.63 | 44.1 | 31.5 | 50 |
